# Evaluating the Dental Caries-Related Information on Brazilian Websites: Qualitative Study

**DOI:** 10.2196/jmir.7681

**Published:** 2017-12-13

**Authors:** Patricia Estefania Ayala Aguirre, Melina Martins Coelho, Daniela Rios, Maria Aparecida Andrade Moreira Machado, Agnes Fátima Pereira Cruvinel, Thiago Cruvinel

**Affiliations:** ^1^ Department of Pediatric Dentistry, Orthodontics and Public Health Bauru School of Dentistry University of São Paulo Bauru Brazil; ^2^ Discipline of Public Health School of Medicine Federal University of Fronteira Sul Chapecó Brazil

**Keywords:** dental caries, internet, consumer health information, health education

## Abstract

**Background:**

Dental caries is the most common chronic oral disease, affecting 2.4 billion people worldwide who on average have 2.11 decayed, missing, or filled teeth. It impacts the quality of life of patients, socially and economically. However, the comprehension of dental caries may be difficult for most people, as it involves a multifactorial etiology with the interplay between the tooth surface, the dental biofilm, dietary fermentable carbohydrates, and genetic and behavioral factors. Therefore, the production of effective materials addressed to the education and counseling of patients for the prevention of dental caries requires a high level of specialization. In this regard, the dental caries-related contents produced by laypersons and their availability on the Internet may be low-quality information.

**Objective:**

The aim of this study was to assess the readability and the quality of dental caries-related information on Brazilian websites.

**Methods:**

A total of 75 websites were selected through *Google*, *Bing*, *Yahoo!*, and *Baidu*. The websites were organized in rankings according to their order of appearance in each one of the 4 search engines. Furthermore, 2 independent examiners evaluated the quality of websites using the DISCERN questionnaire and the Journal of American Medical Association (JAMA) benchmark criteria. The readability of the websites was assessed by the Flesch Reading Ease adapted to Brazilian Portuguese (FRE-BP). In addition, the information presented on the websites was categorized as etiology, prevention, and treatment of dental caries. The statistical analysis was performed using Spearman rank correlation coefficient, Mann-Whitney U test, hierarchical clustering analysis by Ward minimum variance method, Kruskal-Wallis test, and post hoc Dunn test. *P*<.05 was considered significant.

**Results:**

The Web contents were considered to be of poor quality by DISCERN (mean 33.48, standard deviation, SD 9.06) and JAMA (mean 1.12, SD 0.97) scores, presenting easy reading levels (FRE-BP: mean 62.93, SD 10.15). The rankings of the websites presented by Google (ρ=−.22, *P*=.08), Baidu (ρ=−.19, *P*=.53), Yahoo! (ρ=.22, *P*=.39), and Bing (ρ=−.36, *P*=.23) were not correlated with DISCERN scores. Moreover, the quality of websites with health- and nonhealth-related authors was similar (*P*=.27 for DISCERN and *P*=.47 for JAMA); however, the pages with a greater variety of dental caries information showed significantly higher quality scores than those with limited contents (*P*=.009).

**Conclusions:**

On the basis of this sample, dental caries-related contents available on Brazilian websites were considered simple, accessible, and of poor quality, independent of their authorship. These findings indicate the need for the development of specific policies focused on the stimulus for the production and publication of Web health information, encouraging dentists to guide their patients in searching for recommended oral health websites.

## Introduction

The delivery of health care is in a rapid transition from a paternalistic approach to a person-centered model [1,2]. This process aims to improve health outcomes by the construction of a shared decision-making process between health professionals and patients [3,4], characterized by the greater involvement of people in the resolutions and actions concerning their own health [5-10]. The effectiveness of this novel model, however, may be harmed by a considerable number of barriers, such as low education, inadequate access to knowledge, and social and economic deprivation [11].

Dental caries is the most common chronic oral disease worldwide [12], affecting 2.4 billion people [13,14] who on average have 2.11 decayed, missing, or filled teeth [15]. Untreated dental caries impact the quality of life of individuals, socially and economically [16,17], being the first cause of toothache (24.3%) and tooth loss (86%) among Brazilian citizens [18,19]. In addition, dental caries is the fourth most expensive oral condition to be treated [20]; consequently, people are increasingly interested in dental caries-related Web information, particularly about its symptoms and therapies [21]. This disease involves a complex multifactorial etiology, with the interplay between the tooth surface, the dental biofilm, dietary fermentable carbohydrates, and genetic and behavioral factors, which requires a high level of specialization for the production of effective materials addressed to the education and counseling of patients [22-24]. In this regard, the availability of inaccurate contents in open electronic sources may augment the risk of consumption of low-quality dental caries-related information, hampering the person-professional relationship [25,26] and raising the chance of health damages [27]. Even considering habitual Web health consumers and adequate literates, who are more predisposed to identify and reject poor quality information, their decisions are still based on empirical features, such as the order of appearance of links in the search engines, the design factors, and the complexity and the style of information [28,29].

Several studies have already assessed the quality of Web information related to different health conditions [30-39]; however, there is no evidence about the quality of dental Web contents available in Brazil. The aim of this study was to assess the readability and the quality of dental caries-related information retrieved on Brazilian websites.

## Methods

### Study Design

This study analyzed the quality of dental caries-related information available on Brazilian websites. After the development of a specific search strategy, the websites were retrieved by *Google Search*, *Yahoo!*, *Bing*, and *Baidu*. Duplicates, nonspecific, inaccessible, and/or scientific links were excluded. The websites were evaluated by 2 independent examiners using the DISCERN questionnaire [40], the Journal of American Medical Association (JAMA) benchmark criteria [41], and the Flesch Reading Ease adapted to Brazilian Portuguese (FRE-BP) [42]. Furthermore, the websites were dichotomized by the nature of their authorship (health- or nonhealth-related authors). Finally, the websites’ identities were determined by cluster analysis in accordance with the combination of their respective contents (etiology, prevention, and/or treatment of dental caries).

### Search Strategy

The search strategy was designed with regard to the relevance of terms employed by the Internet users. Initially, a general query was performed on *Google Search* to confirm the link of Brazilian Portuguese words to dental caries issues. Additional terms automatically generated by the *Keyword Planner* were included in the analysis. The relevance of each one of 56 terms was subsequently examined in *Google Trends* by observing the monthly variation of their search volume index between the years 2004 and 2015, including all categories of Web queries performed in Brazil (Multimedia Appendix 1). After excluding 53 keywords with irrelevant volume searches, the final search strategy was constructed by the association of three terms (*“cárie”+“carie”+“carie dentaria”*), which correspond to synonyms and typos of dental caries written in Brazilian Portuguese.

### Selection of Websites

The websites were selected through the 4 search engines with the largest market share: *Google Search*, *Baidu*, *Yahoo!,* and *Bing* [43]. On March 21, 2016, the searches were performed using a computer connected to the Internet, previously set up by clearing the cookies and history of each browser. Advanced queries were filtered by idiom (Portuguese) and country (Brazil). The retrieved links were ordered in rankings, considering the position of their appearance in each search engine tool.

Subsequently, the websites were accessed and registered using the WebCite [44], an online service that archive the information exactly as it was recovered, avoiding changes and updates for further analysis.

Finally, the websites were dichotomized according to the nature of their authorship in health- and nonhealth-related authors. Websites or blogs developed by dental or medical associations, universities, educational institutions, health companies, or health professionals were classified as health-related authors. All other pages were classified as nonhealth-related authors. Furthermore, the information presented on the websites was categorized as etiology, prevention, and/or treatment of dental caries. The presence or absence of these contents was graphically represented by the software Genesis (version 1.7.7, Graz, Austria), characterizing the identity of each website [45,46].

### The Assessment of Quality of Websites

Two independent examiners (PEAA and MMC) evaluated the quality of websites using the DISCERN questionnaire [40] and the JAMA benchmark criteria [41]. The DISCERN questionnaire is commonly applied to assess the quality of written information on health treatment choices. The instrument is divided into the following 3 sections: (1) reliability of the publication, (2) specific details of the information about treatment choices, and (3) overall quality rating of the document. It consists of 16 questions with 5-level Likert scale, where the score “1” indicates that the criterion was not fulfilled and the score “5” indicates that the criterion was completely satisfied. The total DISCERN score varies between 15 and 80, as the second question must be disregarded when the first question is scored “1.” Typically, only the results of the first and second sections of this instrument are used to qualify the health content of documents, as follows: very poor (15-26), poor (27-38), fair (39-50), good (51-62), and excellent (63-75) [47].

The JAMA benchmark consists of a series of 4 qualitative criteria that refer to the description of the authorship (author’s name, affiliations, and credentials), attribution (effective references of content), currency (presence of dates of posts and updates of information), and disclosure (the statement of any potential conflicts of interest) of websites. For each fulfilled criterion, 1 point is given, with a total score of 0 to 4.

The websites that were divergently qualified by the examiners were reassessed to the achievement of a consensus score.

### Readability Measures

The FRE-BP [40] was used to assess the readability of the websites based on the following formula: FRE-BP=248.835−(84.6×syllables per word)−(1.015×words per sentence). These metrics were calculated using the online tool Readable.io (Readable.io, Bolney, England) [48] through the information of the respective Uniform Resource Locator (URL) of each website. All analyses were performed based on the overall written content downloaded from these links. The reading difficulty of a text is presented according to the following scores: very easy (75-100), easy (50-75), difficult (25-50), and very difficult (0-25).

### Statistical Analysis

Data were analyzed with the Statistical Package for Social Science (version 21.0; SPSS, Chicago, USA). Although the hypothesis of normal distribution of data was not confirmed by the Kolmogorov-Smirnov test, the statistical analysis was performed by the application of nonparametric tests. The internal consistency of DISCERN was determined by Cronbach alpha. The interrater reliability of DISCERN and JAMA scores provided by the independent examiners was assessed by intraclass correlation coefficient (ICC) for the absolute concordance. The correlations between distinct measures were demonstrated by the Spearman rank correlation coefficients. The significant differences between the dichotomized natures of websites were observed by Mann-Whitney U test. The clusters that emerged from the similarity of websites’ identities were determined by the hierarchical clustering analysis using the Ward minimum variance method. Distinct clusters were compared by Kruskal-Wallis test and post hoc Dunn test. *P* values of <.05 were considered significant for all analyses.

## Results

### Websites

**Figure 1:**
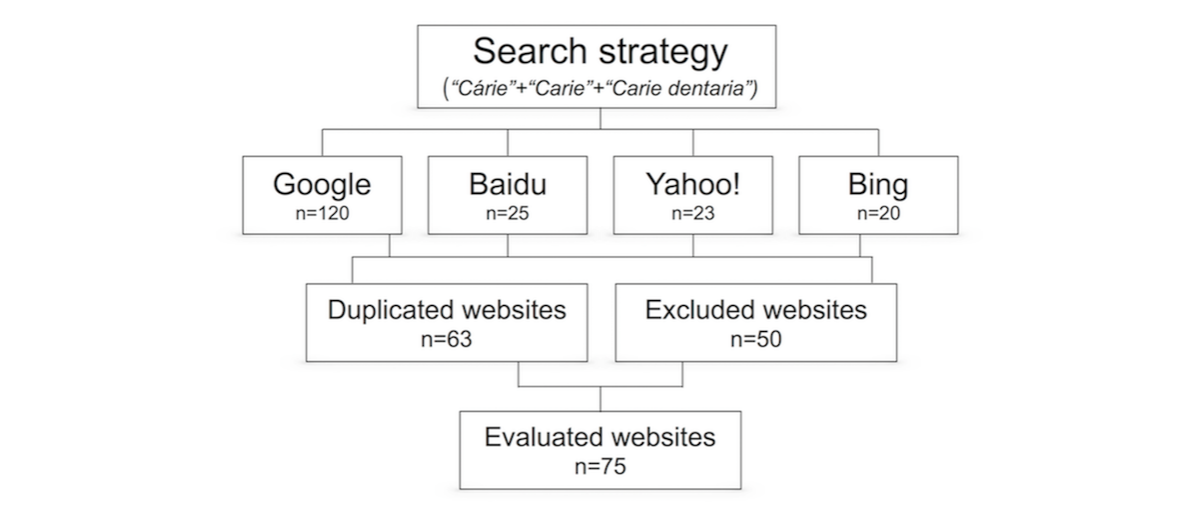
Flowchart depicting the systematic selection of dental caries-related Brazilian websites.

A total of 188 websites were obtained through the first links sequentially retrieved from *Google Search* (n=120), *Baidu* (n=25), *Yahoo!* (n=23), and *Bing* (n=20). Duplicates (n=63), nonspecific websites (n=21), inaccessible links (n=7), and scientific contents (n=22) were excluded. A total of 75 websites met the inclusion criteria for the analysis, as shown in Figure 1. As duplicates were also excluded sequentially, there was a great predominance of *Google Search* links among those that were effectively evaluated, as follows: *Google Search* (n=66), *Baidu* (n=1), *Yahoo!* (n=7), and *Bing* (n=1).

### Reliability of Instruments

The instrument DISCERN displayed an excellent internal consistency (Cronbach alpha=.901), with values of Cronbach alpha varying between .884 and .904 if an item was deleted. A good absolute concordance level was observed between the examiners for the application of the instruments DISCERN questionnaire (ICC=0.73, 95% CI 0.38-0.86) and JAMA benchmark (ICC=0.72, 95% CI 0.56-0.82).

### DISCERN, JAMA, and FRE-BP Scores

The DISCERN and JAMA scores for each website are depicted in the Multimedia Appendix 2. The contents of the websites were classified as of poor quality in accordance with both scores (Table 1), which were significantly correlated (ρ=.58, *P*<.001) (Table 2). In addition, the sum of partial DISCERN scores (sections 1 and 2) was strongly correlated with the scores of section 3 (ρ=.92, *P*<.001). A total of 20 websites scored ≥39, with a mean overall quality of 3.71. A digital encyclopedia opened to public contributors (“ *Wikipedia* ”) and a page specialized in dental health information (“ *ident* ”) showed the two highest DISCERN scores (>60). Only 9% (7/75) of the websites presented at least three required information displayed by JAMA benchmark criteria. The minor percentage of pages presented authorship (36%, 27/75), attribution (18.7%, 14/75), currency (17.3%, 13/75), and disclosure (40%, 30/75).

According to the FRE-BP scores, the websites were considered simple and accessible for most population (Table 1). In addition, the difficulty level in reading of websites was weakly and negatively correlated with DISCERN scores (Table 2). The ranking of the websites in the 4 engines was not correlated with DISCERN and FRE-BP scores. Distinctly, JAMA benchmark scores showed a weakly negative correlation with the ranking of the websites retrieved from *Google Search* (Table 2).

The scores of websites with health- and nonhealth-related authors were statistically similar, considering DISCERN (*P*=.29) and JAMA benchmark (*P*=.47) (Table 3). Nevertheless, the written documents produced by health-related authors were considered significantly more difficult than their counterparts.

### Websites’ Identities

**Figure 2:**
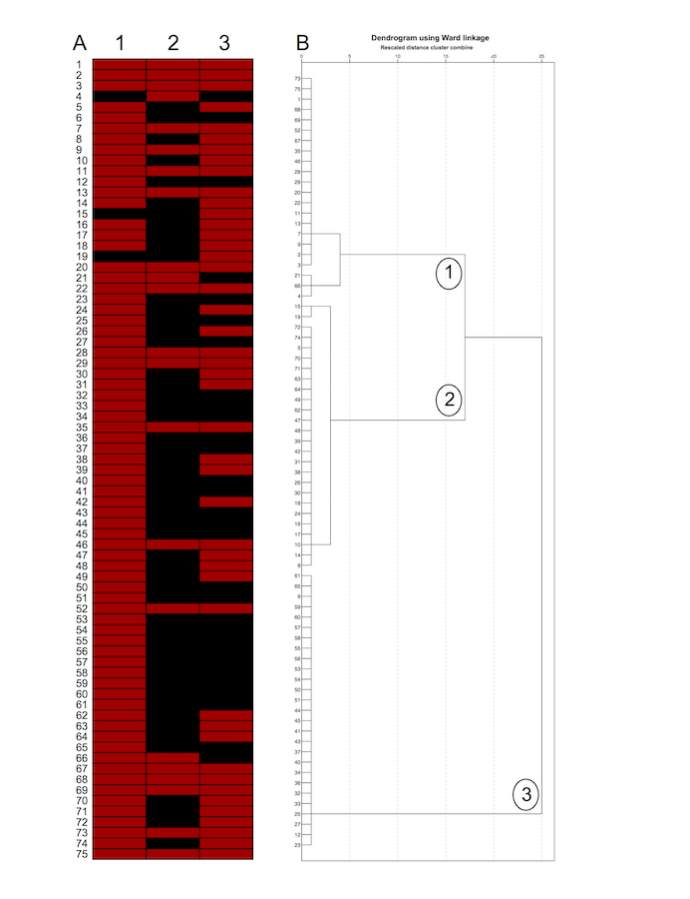
Cluster analysis of the websites. (A) The representation of websites’ IDs regarding the content of information: etiology (1), treatment (2), and/or prevention (3) of dental caries. Red and black bars mean the presence and absence of the type of information, respectively. (B) Dendrogram depicts three clusters originated from the websites’ IDs (hierarchical clustering analysis by Ward’s minimum variance method).

The hierarchical clustering analysis yielded three distinct websites’ identities, as shown in Figure 2. Overall, websites containing contents of all 3 categories (cluster 1) showed higher quality scores than websites containing contents of only 1 (cluster 3) or 2 (cluster 2) categories. The DISCERN scores of cluster 1 were statistically higher than those of cluster 3 (*P*=.009) (Table 4). Additionally, the percentages of websites with health-related authors were 40.9% for cluster 1, 61.5% for cluster 2, and 48.1% for cluster 3.

**Table 1:** Descriptive statistics of scores of DISCERN, the Journal of American Medical Association benchmark, and Flesch Reading Ease adapted to Brazilian Portuguese.

Outcomes	S1^a^	S2^a^	S3^a^	DISCERN (S1+S2)	JAMA^b^	FRE-BP^c^
Mean (SD)	18.89 (5.70)	14.59 (5.40)	2.20 (0.85)	33.48 (9.06)	1.12 (0.97)	62.93 (10.15)
Median	18.00	14.00	2.00	33.00	1.00	63.56
Minimum	8.00	7.00	1.00	18.00	0.00	37.98
Maximum	34.00	28.00	4.00	60.00	3.00	88.23

^a^S1, S2, and S3: 3 different sections of DISCERN.

^b^JAMA: the Journal of American Medical Association.

^c^FRE-BP: Flesch Reading Ease adapted to Brazilian Portuguese.

**Table 2:** Cross correlation between the ranking of websites presented in the 4 engines, DISCERN, the Journal of American Medical Association benchmark, and Flesch Reading Ease adapted to Brazilian Portuguese.

Outcomes	Ranking Baidu (ρ, *P*)	Ranking Yahoo! (ρ, *P*)	Ranking Bing (ρ, *P*)	DISCERN (S1+S2^a^; ρ, *P*)	JAMA^b^ (ρ, *P*)	FRE-BP^c^ (ρ, *P*)
Ranking Google	.78 .02	.86 <.001	.78, .02	−.22, .08	−.28, .02	.03, .84
Ranking Baidu		.60, .40	.95, <.001	−.19, .53	−.11, .73	.02, .96
Ranking Yahoo!			.77, .07	.22, .39	−.02, .93	−.04, .89
Ranking Bing				−.36, .23	−.19, .54	.03, .92
DISCERN					.58, <.001	−.23, .05
JAMA						.04, .76

^a^S1 + S2 = sum of scores of sections 1 and 2 of DISCERN.

^b^JAMA: the Journal of American Medical Association.

^c^FRE-BP: Flesch Reading Ease adapted to Brazilian Portuguese.

**Table 3:** Descriptive statistics of websites with health- and nonhealth-related authors for DISCERN, the Journal of American Medical Association benchmark, and Flesch Reading Ease adapted to Brazilian Portuguese.

Websites		S1^a^	S2^a^	S3^a^	DISCERN (S1+S2)	JAMA^b^	FRE-BP^c^
**Health-related authors (n=38)**							
	Mean (SD)^d,e^	19.58 (5.76)	14.68 (5.02)	2.34 (0.82)	34.26 (8.85)	1.05 (1.01)	63.26 (10.15)
	Median	18.00	14.00	2.00	33.00	1.00	63.87
	Minimum	11.00	7.00	1.00	18.00	0.00	37.98
	Maximum	34.00	26.00	4.00	58.00	3.00	88.23
**Nonhealth-related authors (n=37)**							
	Mean (SD)^d,e^	18.19 (5.76)	14.49 (5.75)	2.05 (0.88)	32.68 (9.33)	1.19 (0.94)	59.75 (10.34)
	Median	18.00	15.00	2.00	33.00	1.00	62.55
	Minimum	8.00	7.00	1.00	18.00	0.00	46.24
	Maximum	33.00	28.00	4.00	60.00	3.00	72.73

^a^S1, S2, and S3: 3 different sections of DISCERN.

^b^JAMA: the Journal of American Medical Association.

^c^FRE-BP: Flesch Reading Ease adapted to Brazilian Portuguese.

^d,e^Significant statistical differences between the groups (Mann-Whitney *U* test, *P*<.05).

**Table 4:** Descriptive statistics of different clusters of websites for DISCERN, the Journal of American Medical Association benchmark, and Flesch Reading Ease adapted to Brazilian Portuguese.

Cluster		S1^a^	S2^a^	S3^a^	DISCERN	JAMA^b^	FRE-BP^c^
**1 (n=22)**							
	Mean (SD)^d,e,f^	19.05 (5.64)	18.05 (6.26)	2.41 (1.01)	39.50 (11.41)	1.05 (1.00)	60.04 (10.87)
	Median	18.50	17.50	2.50	38.50	1.00	57.03
	Minimum	8.00	9.00	1.00	22.00	0.00	41.23
	Maximum	32.00	28.00	4.00	64.00	3.00	78.86
**2 (n=26)**							
	Mean (SD)^d,e,f^	18.85 (4.65)	15.50 (3.78)	2.35 (0.75)	36.69 (8.19)	1.19 (0.85)	65.61 (7.80)
	Median	18.00	16.00	2.00	35.00	1.00	66.03
	Minimum	12.00	8.00	1.00	22.00	0.00	48.57
	Maximum	34.00	24.00	4.00	62.00	3.00	80.28
**3 (n=27)**							
	Mean	18.81^d^	10.89^e^	1.89^d^	31.59^f^	1.11^d^	62.69^d^
	SD^g^	6.82	3.33	0.75	8.69	1.09	11.15
	Median	16.00	11.00	2.00	29.00	1.00	63.06
	Minimum	11.00	7.00	1.00	19.00	0.00	37.98
	Maximum	33.00	19.00	3.00	47.00	3.00	88.23

^a^S1, S2, and S3: 3 different sections of DISCERN.

^b^JAMA: the Journal of American Medical Association.

^c^FRE-BP: Flesch Reading Ease adapted to Brazilian Portuguese.

^d,e,f^Significant statistical differences between the groups (Kruskal-Wallis test and post hoc Dunn test, *P*<.05).

## Discussion

### Principal Findings

To the best of our knowledge, this is the first study to assess the quality of dental caries-related information on Brazilian websites. In general, our results showed a predominance of low-quality contents, with a low rate of websites (26.7%) being classified as acceptable to high-quality levels (DISCERN≥39). These results are consistent with the results of similar studies [49,50]; Blizniuk et al [49] demonstrated lack of quality of dental caries-related information on English websites (DISCERN=44), whereas Leite and Correia [50] identified only 4 out of 75 Portuguese dental caries-related websites certified with the Health On the Net Foundation (HON) code, a trustworthy certification granted by a nongovernmental institution that evaluates the quality of health information on the Internet [51]. However, we did not consider the HON code in our methods, as only 1 Brazilian website was certified. Surprisingly, the content of this website was qualified as inadequate by both instruments utilized in this study.

We believe that the assessment of the quality of websites was improved by the simultaneous application of distinct criteria, particularly because they were only fairly correlated. Additionally, the outstanding internal consistency and interrater agreement of DISCERN when employed in these analyses are noteworthy. In contrast, the determination of the internal consistency of the JAMA benchmark is limited because it aims at the elucidation of specific data about technical and editorial production of websites. Although its four elements should be better interpreted individually, we calculated the central tendency measures of the JAMA benchmark to evaluate its relationship with other indicators.

The strict investigation executed by 2 health professionals may be linked to perception biases through the underestimation of the quality of websites; nevertheless, Griffiths and Christensen [52] revealed no significant differences between the scores of DISCERN given by professionals and laypersons. In addition, as cluster-based results were not influenced by the nature of websites’ authorship, the better performance of websites that published topics about etiology, prevention, and treatment of dental caries reflects the significant impact of the completeness of contents on the process of qualification of information. This finding is supported by the study of Diviani et al [29], who showed that the amount of information available on a website influences the perception of improved quality of digital contents by Internet users. No significant differences were found when comparing DISCERN scores with JAMA scores of websites of health- and nonhealth-related authors, suggesting that the quality criteria considered in these instruments were not a concern for the most dentists and/or dental companies during the production of electronic contents.

Although the negative correlation between FRE-BP and DISCERN scores was discrete, this trend should be regarded as an exacerbating factor for the impact of the low quality of information on the Internet users, as it demonstrates that more accessible contents are even worse in quality. This fact raises an important concern with regard to the high percentage of basic literate Brazilian youths (99%) and adults (93%) [53], as the understanding of medical information probably requires more advanced educational abilities [28]. In this context, the shared decision-making process could be deteriorated by the misunderstanding of health information and the development of harmful health beliefs.

**Design considerations**

According to the Vital Signs report [54], there are 4 types of individuals with regard to health care situation: (1) those who agree and accept the treatment decision, (2) those who access the Web to confirm the diagnosis given by a professional, (3) those who are involved in the decision-making process, and (4) those who are in complete control of their treatment relying on the information found by themselves. In this context, although health professionals are still considered the most important source of health advice, the easier and more affordable access to the Internet predisposes people to seek health counseling online [29]. Furthermore, 65% of health seekers frequently begin their searches using an engine bar instead of looking for information on specific portals [54]. Consequently, millions of health-related queries are entered in *Google Search* daily [55]; therefore, the methodological approach adopted for the construction of our search strategy probably improved the chances of retrieving the websites in a similar way to that usually performed by netizens.

To evaluate the correlation of the order of appearance of links in the search engines with the quality of their contents, we assessed a quite larger number of websites than the Internet users could be interested in [56]. For instance, Google’s PageRank uses more than 200 factors based on Larry Page’s algorithms to order the links by their relevance from the query [57]. In this study, the links found on the Google’s first page were represented by 5 blogs, 2 dental clinics, and 1 commercial website. Their contents were alarming, for example, with the description of *dental treatments without dentists*. In addition, the order of appearance of the websites was not correlated with DISCERN scores in the 4 different engines, that is, the algorithms created to retrieve the links associated with dental caries seemed to not have any relationship with the quality of available information, which may contribute even more with the deterioration of the health education process.

**Limitations**

This study presents some limitations. First, although laypersons could be interested in reading more specialized documents, the links associated to scientific publications, such as papers and books, were not considered into our analyses. This decision was based on two main reasons: (1) the DISCERN was developed to assess the quality of information presented to health seekers, that is, its application would be inadequate and unproductive to analyze scientific contents; and (2) probably, the great specificity and the technical language of scientific papers lead people to look for documents that explore more general knowledge about the disease. Second, it was not feasible to evaluate other types of website media, such as figures, films, and podcasts, as the DISCERN was specifically developed to assess the quality of written documents. Finally, the unknown audience of the websites prevented the determination of the impact of each source on the diffusion of dental knowledge, although our results were supposedly obtained from the most accessed websites, considering that they were the first dental caries-related links retrieved by search engines.

**Challenges**

The development of specific regulations in this field is arduous, requiring an intense debate to avoid the suppression of the rights of freedom of expression and opinion [58]. Likewise, it is almost impossible to control the publication of Web contents, particularly because of the diffusion of personal opinion contained in health blogs. The use of codes and seals for the certification of websites could be a good approach to indicate the useful health information, particularly if the accreditation is based on rigorous criteria and if the Internet users can easily view the certification on the website. Nevertheless, the continuous consumption of misleading knowledge could deteriorate the person-dentist relationship; hence, the professional should be prepared to face this challenge effectively, advising and encouraging their patients to explore information on recommended websites, warning people about the risks of health home practices, and contributing to the production of good quality electronic materials.

**Conclusions**

In conclusion, regarding the present sample of Brazilian websites, dental caries-related contents were considered simple, accessible, and of poor quality based on the results of FRE-BP, DISCERN, and JAMA benchmark scores, respectively. This pattern does not seem to rely on the natures of websites’ authorship but on the multiplicity of categories of information that they covered. These findings indicate the need for the development of special policies focused on the stimulus for the production and publication of Web health information, encouraging dentists to guide their patients to search for recommended oral health websites.

## Appendix

Multimedia Appendix 1 List of 56 dental caries-related keywords retrieved in Google Search and Keyword Planner (written in Brazilian Portuguese).

Multimedia Appendix 2 List of websites and respective identities, ranking, DISCERN, and JAMA benchmark scores.

## Abbreviations

FRE-BP: Flesch Reading Ease adapted to Brazilian Portuguese
HON: Health On the Net Foundation
ICC: interclass correlation coefficient
JAMA: the Journal of American Medical Association
