# Supplementary material for: Evaluating the Dental Caries-Related Information on Brazilian Websites: Qualitative Study
Source: J Med Internet Res. 2017 Dec 13;19(12):e415. doi: 10.2196/jmir.7681 (PMC5745348; doi:10.2196/jmir.7681)
Supplement: Multimedia Appendix 1 [file jmir_v19i12e415_app1.pdf]

Appendices

Multimedia Appendix 1: List of 56 dental caries-related keywords retrieved in *Google Search* and *Keyword Planner* (written in Brazilian Portuguese)

Cárie dentária	Carie	Carie sintomas
Carie dentaria	Cárie no dente	Causa cárie
Dentes cariados	Carie no dente	Causa carie
Cárie sintomas	Cárie de dente	Prevenção de cárie
Carie sintomas	Carie de dente	Prevenção de carie
Cárie de mamadeira	Dente com cárie	Cárie tratamento
Carie de mamadeira	Dente com carie	Carie tratamento
Dente cariado	Cárie dente	Tratar cárie
Cárie em dente	Carie dente	Tratar carie
Carie em dente	Cárie dente de leite	Cárie profunda
Cárie dental	Carie dente de leite	Carie profunda
Carie dental	Lesão cariosa	Tirar cárie
Cárie	Cárie sintomas	Tirar carie
Ponto preto no dente	Detecção de carie	Dente estragado
Buraco no dente	Furo no dente	Cáries em crianças
Dente furado	Cáries na infância	Cáries precoce da infância
Mancha preta no dente	Dente podre	Mancha branca nos dentes
Bicho no dente	Furinho no dente	
Dente com cárie	Cáries do biberão	
