# Supplementary material for: Evaluating the Dental Caries-Related Information on Brazilian Websites: Qualitative Study
Source: J Med Internet Res. 2017 Dec 13;19(12):e415. doi: 10.2196/jmir.7681 (PMC5745348; doi:10.2196/jmir.7681)
Supplement: Multimedia Appendix 2 [file jmir_v19i12e415_app2.pdf]

Multimedia Appendix 2: List of websites and respective identities, ranking, DISCERN and JAMA benchmark scores

ID	Ranking	Webcite© links	DISCERN	JAMA
1	1	http://www.webcitation.org/6gAssu22A	27	1
2	4	http://www.webcitation.org/6gAszf6Ya	54	2
3	5	http://www.webcitation.org/6gAt8Th7T	46	2
4	6	http://www.webcitation.org/6gAt9Lrow	22	0
5	8	http://www.webcitation.org/6gAtAGfc5	29	3
6	9	http://www.webcitation.org/6gAtJtqBf	29	1
7	10	http://www.webcitation.org/6gAtK9Hkx	28	2
8	11	http://www.webcitation.org/6gAtUCdLk	22	0
9	12	http://www.webcitation.org/6gAtUUbOc	38	2
10	13	http://www.webcitation.org/6gAtUjjpS	34	1
11	15	http://www.webcitation.org/6gAtav5BA	30	0
12	18	http://www.webcitation.org/6gAtbErYI	43	3
13	20	http://www.webcitation.org/6gAtojv8r	23	0
14	23	http://www.webcitation.org/6gAtpPO6c	43	2
15	24	http://www.webcitation.org/6gAtpZQVN	35	2
16	25	http://www.webcitation.org/6gAuCOnjX	43	1
17	26	http://www.webcitation.org/6gAu5qIze	33	1
18	27	http://www.webcitation.org/6gAuFvS95	35	2
19	28	http://www.webcitation.org/6gAubHtOy	52	2
20	29	http://www.webcitation.org/6gAubZIvp	55	2
21	30	http://www.webcitation.org/6gAucjtlE	44	1
22	31	http://www.webcitation.org/6gAukt1zx	53	2
23	32	http://www.webcitation.org/6gAv4y0BP	26	0
24	33	http://www.webcitation.org/6gAv5uAe0	32	1
25	34	http://www.webcitation.org/6gAvEe4M3	27	0
26	35	http://www.webcitation.org/6gAvFBPGY	33	1
27	36	http://www.webcitation.org/6gAvPf181	38	1
28	37	http://www.webcitation.org/6gAvbJoIa	33	2
29	38	http://www.webcitation.org/6gAvnme4x	39	0
30	39	http://www.webcitation.org/6gCNJxpyQ	35	1
31	40	http://www.webcitation.org/6gCNUA2Zf	62	3
32	41	http://www.webcitation.org/6gCNUQl1H	43	2
33	42	http://www.webcitation.org/6gCNYnUcQ	47	3
34	43	http://www.webcitation.org/6gCNiSeRG	24	0
35	44	http://www.webcitation.org/6gCNlfQun	44	1
36	45	http://www.webcitation.org/6gCNvAUE2	46	2
37	46	http://www.webcitation.org/6gCO37i9v	47	3
38	47	http://www.webcitation.org/6gCO3HEtI	42	2
39	48	http://www.webcitation.org/6gCO3OdWH	26	0
40	49	http://www.webcitation.org/6gCPyhQyD	41	3
41	50	http://www.webcitation.org/6gCPyrck1	35	2
42	51	http://www.webcitation.org/6gCQ5kGPk	35	1
43	52	http://www.webcitation.org/6gCQ5sJK7	25	1

44	53	http://www.webcitation.org/6gCQ61Crc	35	1
45	54	http://www.webcitation.org/6gCQHdyA2	21	0
46	55	http://www.webcitation.org/6gCQKg5QM	38	2
47	56	http://www.webcitation.org/6gCQKpFZj	37	1
48	57	http://www.webcitation.org/6gCQRmTFz	34	0
49	58	http://www.webcitation.org/6gCQaLGNj	36	1
50	59	http://www.webcitation.org/6gCQaV7BJ	28	0
51	60	http://www.webcitation.org/6gCQac813	37	1
52	61	http://www.webcitation.org/6gCQt800K	39	0
53	62	http://www.webcitation.org/6gCQtDHZz	22	1
54	63	http://www.webcitation.org/6gCQtKyoa	34	1
55	64	http://www.webcitation.org/6gCR3KMa1	19	2
56	65	http://www.webcitation.org/6gCRLFpG6	22	0
57	66	http://www.webcitation.org/6gCRLMPz3	29	1
58	67	http://www.webcitation.org/6gCRXzWtD	32	2
59	68	http://www.webcitation.org/6gCRY6VUk	29	0
60	69	http://www.webcitation.org/6gCRfqghB	28	0
61	70	http://www.webcitation.org/6gCRYWA6g	19	0
62	71	http://www.webcitation.org/6gCRfyE1x	40	1
63	72	http://www.webcitation.org/6gCRlar4K	31	1
64	73	http://www.webcitation.org/6gCRy27Ro	29	0
65	74	http://www.webcitation.org/6gCRyCLjT	27	0
66	75	http://www.webcitation.org/6gCS4jqA6	28	0
67	21	http://www.webcitation.org/6hGaF3NKE	45	0
68	2	http://www.webcitation.org/6hGbGKOK4	52	0
69	3	http://www.webcitation.org/6hGbJu0jN	64	3
70	7	http://www.webcitation.org/6hGbTSraL	39	2
71	14	http://www.webcitation.org/6hGbxGKvz	41	1
72	16	http://www.webcitation.org/6hGc0ogdT	45	1
73	19	http://www.webcitation.org/6hGcCMSLW	30	0
74	22	http://www.webcitation.org/6hGcL60Ya	31	0
75	17	http://www.webcitation.org/6hGXUeroU	37	1
